# DIFTOS: A Distributed Infrastructure-Free Traffic Optimization System Based on Vehicular Ad Hoc Networks for Urban Environments

**DOI:** 10.3390/s18082567

**Published:** 2018-08-06

**Authors:** Weidong Zhang, Nyothiri Aung, Sahraoui Dhelim, Yibo Ai

**Affiliations:** 1 National Center for Materials Service Safety, University of Science and Technology Beijing, Beijing 100083, China; ybai@ustb.edu.cn; 2 School of Computer and Communication Engineering, University of Science and Technology Beijing, Beijing 100083, China; nyothiriaung@xs.ustb.edu.cn (N.A.); sahraoui.dhelim@xs.ustb.edu.cn (S.D.)

**Keywords:** congestion avoidance, traffic optimization, congestion avoidance, VANET, intelligent transportation systems, ITS, path planning, distributed server

## Abstract

Aiming to alleviate traffic congestion, many congestion avoidance and traffic optimization systems have been proposed recently. However, most of them suffer from three main problems. Firstly scalability: they rely on a centralized server, which has to perform intensive communication and computational tasks. Secondly unpredictability: they use smartphones and other sensors to detect the congested roads and warn upcoming vehicles accordingly. In other words, they are used to solve the problem rather than avoiding it. Lastly, infrastructure dependency: they assume the presence of pre-installed infrastructures such as roadside unit (RSU) or cellular 3G/4G networks. Motivated by the above-mentioned reasons, in this paper, we proposed a fully distributed and infrastructure-less congestion avoidance and traffic optimization system for VANET (Vehicular Ad-hoc Networks) in urban environments named DIFTOS (Distributed Infrastructure-Free Traffic Optimization System), in which the city map is divided into a hierarchy of servers. The vehicles that are located in the busy road intersections play the role of servers, thus DIFTOS does not rely on any centralized server and does not need internet connectivity or RSU or any kind of infrastructure. As far as we know, in the literature of congestion avoidance using VANET, DIFTOS is the first completely infrastructure-free congestion avoidance system. The effectiveness and scalability of DIFTOS have been proved by simulation under different traffic conditions.

## 1. Introduction

Due to recent fast urbanization, many large cities around the world are suffering from an unprecedented rise in traffic congestion. According to a recent urban transportation report, the economic loss caused by traffic congestion in the U.S. in terms of both fuel consumption and travel time delay was estimated at $121 billion in 2011 and is expected to reach $199 billion in 2020 [1]. Not to mention highly populated countries like China and India. Without a doubt, traffic congestion is one of the most prominent problems that scientific and industrial communities are trying to solve today.

In the literature of Intelligent Transportation Systems (ITS), the problem of finding the optimal path for a given vehicle is still under investigation, many congestion avoidance and traffic optimization systems have been proposed but most of them suffer from several drawbacks. Google Maps, Baidu Maps, and TomTom are the leading industrial companies in real-time traffic optimization. They provide real-time traffic conditions and route planning services, based on Global Positioning System (GPS), they measure the vehicle’s speed, and eventually detect the congestion if a group of adjacent vehicles has very low speed at a specific road. This approach suffers from two drawbacks, firstly, it cannot prevent the congestion before it happens. Secondly, it cannot make a quick response to emergency cases such as traffic accidents.

The current industrial solutions for GPS navigation are divided into two groups, either dedicated navigators (e.g., TomTom [2] and uNAVI [3]) or smartphone based navigator (e.g., Waze [4] and Skobbler [5]). Most of them are using the shortest travel delay path algorithm based on road traffic statistics or real-time road traffic measurement. There are two major drawbacks of using such systems, (1) the routes that they provide are individually optimized paths for each vehicle. That is, they do not consider the collaboration among vehicles in congested road areas for better navigation; (2) the congestion is detected based on vehicles’ speed in different road segments. A road segment is considered as congested if the mean speed of the passing vehicles has been reduced to an abnormal level. In other words, these systems are detecting the congestion rather than preventing it from happening in the first place.

Alternatively, the emerging Vehicular Ad-hoc Networks (VANET), that support both, Vehicle-to-Vehicle (V2V) and Vehicle-to-Infrastructure (V2I) wireless communication, can be used in many traffic related applications. Such as traffic congestion estimation [6], autonomous car parking system [7], traffic flow prediction [8], CO_2_ emissions reduction [9], emergency situations management [10,11] and route discovery [12]. However, deploying a new infrastructure, such as Roadside Units (RSU), in a big city like Beijing for instance, could be a quite costly task. In addition to the high cost, in case of infrastructure failure due to natural disaster, say an earthquake for instance, the vehicles must be able to coordinate the evacuation of critical areas in a fast and orderly way, this requires the ability to effectively communicate with each other without the need to rely on any external infrastructure.

Motivated by the above-mentioned reasons, in this paper we proposed a new distributed infrastructure-less congestion prediction and traffic optimization system for VANET in urban environment named DIFTOS. The city map is divided into a hierarchy of servers. The vehicles that are located in the busy road intersections form a virtual vehicular server (VVS), thus DIFTOS does not rely on any centralized server and does not need cellular network connectivity, RSU or any kind of infrastructure.

The contributions of this work are summarized as follows:

- DIFTOS is completely infrastructure-less and does not rely on any other network (cellular network, RSU), and it is totally distributed and does not rely on a centralized server.
- DIFTOS is designed in a hierarchical fashion, thus short length path requests are resolved locally, and does not need to travel long distances.
- To demonstrate the validity of DIFTOS, we have conducted a thorough comparison with existing approaches.

The rest of the paper is organized as follows: Section 2 highlights recent related works, In Section 3, we present the proposed system’s architecture. Whereas Section 4 sets up the theoretical foundation of DIFTOS. Furthermore, Section 5 gives details about the simulation environment. While in Section 6 we discuss the performance evaluation of DIFTOS and analyze the obtained results. Finally, we conclude the paper and present the future directions in Section 7.

## 2. Related Works

Many congestion avoidance systems using VANET have been proposed recently; Wang et al. [13] proposed a real-time path-planning algorithm, where a stochastic Lyapunov optimization technique is exploited to improve the overall spatial utilization of a road network and reduce the average vehicle travel cost for avoiding vehicles from getting stuck in congestion. A vehicle rerouting system called Next Road Rerouting (NRR) is proposed in [14], where a heuristic rerouting decision is made upon a cost function that takes into account the driver’s destination and local traffic conditions. Jeong et al. [15], proposed SAINT, a cloud-based vehicular traffic optimization system, in which the vehicles report their navigation experiences and travel paths to the vehicular cloud, thus, the vehicular cloud can know real-time road traffic conditions and vehicle trajectories; the vehicular cloud uses a mathematical model to calculate road segment congestion estimation for global traffic optimization. Pan et al. [16] proposed DIVERT, a distributed vehicular re-routing system for congestion avoidance. DIVERT offloads a large part of the re-routing computation to the vehicles, and thus, the re-routing process becomes practical in real-time. To take collaborative re-routing decisions, the vehicles exchange messages over VANET. However, DIVERT is not an infrastructure-less system because it still uses a server and internet connectivity to determine an accurate global view of the traffic. The work in [17] presented RoadRunner, a congestion control system implemented as an Android app that relies on LTE network and V2V communications. The proposed system distributes tokens to vehicles as permission for their entry into roads; tokens are provided and managed by a centralized server.

However, the systems presented in [13,14,15,16,17], assume the existence of pre-installed infrastructures, such as RSU or cellular network. As far as we know, DIFTOS is the first system that is completely infrastructure-free and does not rely on any existing infrastructure such as RSU or cellular network. In Table 1 a comparison between DIFTOS and state-of-the-art systems is presented.

Besides the traffic congestion avoidance systems, many works have used VANETs for traffic related applications. Aldegheishem et al. [18] developed a new protocol, named as the Traffic Accidents Reduction Strategy (TARS), for VANET to minimize the number of road accidents, and decrease the death rate caused by road accidents. The simulation results showed that their proposed scheme has outperformed DBSR and POVRP routing protocols. Sam et al. [19] presented a novel scheme to select important safety messages for verification. The proposed scheme uses location and direction of the sender, as well as proximity and relative-time between vehicles, to reduce the number of irrelevant messages verified (i.e., messages from vehicles that are unlikely to cause an accident). Hu et al. [20] proposed a link Lifetime-aware Beacon-less Routing Protocol (LBRP) that is designed for large volume content delivery in VANETs. Each vehicle makes the forwarding decision based on the message header information and its current state, including the speed and position information. A semi-markov process analytical model is proposed to evaluate the expected delay in constructing one routing path for LBRP. While Garcia-Lozano et al. [21] discussed the applicability of several options to avoid the broadcast storm problem while trying to achieve the maximum coverage of the region of interest. Specifically, they have evaluated through simulations different ways to detect and take advantage of intersections and a strategy based on store-carry-forward to overcome short disconnections between groups of vehicles. García-Campos et al. [22] presented a methodology for conducting reliable simulations of routing protocols in VANETs urban scenarios. The proposed methodology includes relevant simulation aspects such as measurement period, selection of source-destination pairs for the communication traffic flows, number of simulations, mobility models based on road city maps, performance metrics and different analyses to evaluate routing protocols under different conditions.

## 3. System Architecture

In this section, we present the architecture of the proposed system:

### 3.1. Assumptions

- All vehicles are equipped with a dedicated short-range communication (DSRC) device.
- All vehicles are equipped with a GPS-based navigation system that has a digital roadmap.
- All communications are done using multi-hop vehicle-to-vehicle communication model (V2V).
- Drivers input the destination into their GPS-based navigation system as a navigation request when they start traveling. The VVS computes the shortest non-congested path and reserves it for them.
- Vehicles report the updates about their travel experience in road segments and at intersections along their travel path to their corresponding VVS.
- Since the travel paths are very sensitive information, all the communications between DIFTOS-Client and DIFTOS-Server are encrypted.
- The road segments are reserved in the order of a first come first served policy.

### 3.2. System Components

DIFTOS has two main components, DIFTOS-Client and DIFTOS-Server. All the vehicles are equipped with these two components, but they are used in different contexts.

#### 3.2.1. DIFTOS-Client

**Figure 1:**
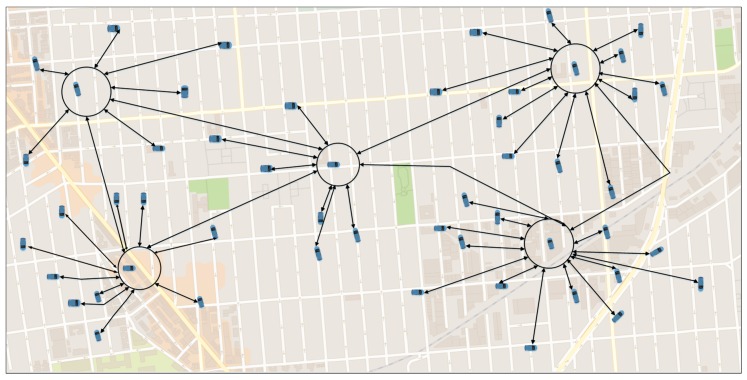
Clients-VVS communications.

This component is used when the vehicle wants to go to a specific position in the map; the DIFTOS-Client of that vehicle sends a path request to the VVS of the corresponding region, aiming to get the fastest (least congested) path to its destination. As shown in Figure 1, each vehicle solicits the nearest VVS (denoted by circles in Figure 1) to get the least congested path to its destination.

#### 3.2.2. DIFTOS-Server

The vehicles situated in the VVS area (typically busy intersections), collaboratively elect a vehicle that will play the role of the server, the DIFTOS-Server procedure will be executed in that vehicle. The server vehicle maintains the road reservations matrix (RRM), which contains the weight of all the road segments situated in the coverage of current VVS in different time slots.

### 3.3. Virtual Vehicular Server

As mentioned above, the servers are distributed as a hierarchy.

#### 3.3.1. Hierarchical Partitioning

**Figure 2:**
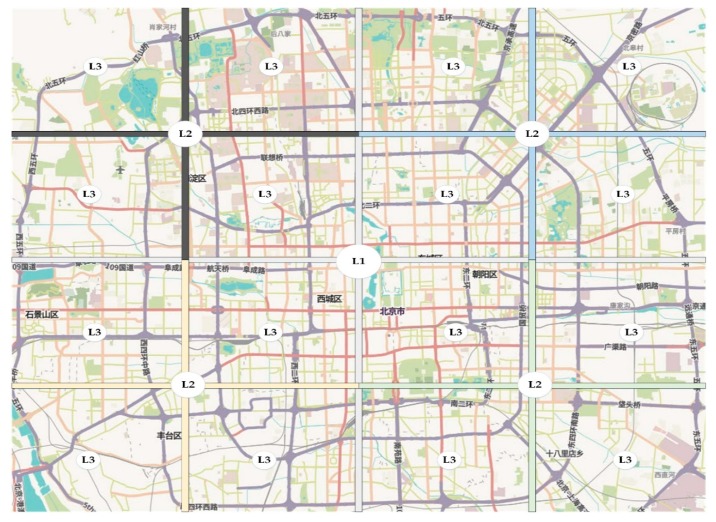
VVS hierarchical partition.

The busiest intersection near the center of the city map is chosen as the highest level VVS (denoted as L1 in Figure 2), level 1 server covers the whole map. The map is divided into four regions, in each region, there is level-two server (denoted as L2 in Figure 2), level-two server is responsible only for that region, not the whole map. Each level-two region is further divided into four level-three regions, and so on and so forth. In Figure 2 we have used three levels hierarchy just as an example; the number of hierarchy levels can be adjusted according to the size of the city. To avoid overloading the upper levels servers, each path request is treated based on its scope, more specifically it is treated at the lowest level server that covers the source and destination positions, thus short paths that are within the scope of level 3 or level 2 servers are treated locally.

#### 3.3.2. Server Selection

The VVS zones are typically chosen as intersections, to ensure the continuous presence of vehicles that will serve as DIFTOS-Server. When a vehicle enters a VVS region and stops at traffic lights, it announces itself as a server, by broadcasting a “Server_Candidate” message to a nearby vehicle and initialize a wait response timer, in case a “Server_Exist” message is received before timer termination, the candidate will stop assuming itself as a server. Otherwise, it will broadcast “Server_Elected” message to nearby vehicles, and start doing server responsibility, which is to resolve path requests and update the RRM. When the server leaves the VVS region, it forwards the RRM to one of the nodes that are waiting for the traffic light and announce it as a server by broadcasting “Server_Replaced” message.

It is worthwhile to mention that in high density scenarios, the server selection scheme might suffer from broadcast storm problem [21]. To alleviate the effects of broadcast storm problem, the server selection in DIFTOS includes a broadcast suppression scheme. The latter is based on two techniques. (1) Number of hops: In which the number of hops is limited to prevent the vehicles from retransmitting the message to vehicles that already received it; (2) Transmitting location: The vehicles check whether they are still inside the region of interest (VVS region) before retransmitting the message.

## 4. System Modelling

In this section, we will explain the modeling details of DIFTOS.

### 4.1. Notations

For the sake of readability, in Table 2 we have listed all the notations that are used in this paper.

### 4.2. Road Network Graph

The city map is represented as graph data structure. Let road network graph (RNG) be a directed graph G=(I,Ω,V) that represents the map of a given city, where *I* is the set of vertices (i.e., intersections), and Ω is the set of directed edges $r_{i,j}$ (i.e., road segments) for i,j∈I, each road segment connects two adjacent intersections. Except for some special cases, most of the road segments are bidirectional with possible multiple lanes in each direction, road segment between two adjacent intersections i and j has two different road segments with opposite directions, i.e., road segment $r_{i,j}$ and road segment $r_{j,i}$. Finally, V is the set of all vehicles on the city map.

### 4.3. Traffic Flow and Travel Delay

In DIFTOS, time is represented as a set of successive time slots, $T=\left\{t_{1},t_{2},\ldots ,t_{n}\right\}$, we have used the flow speed on the road link within the time slots to indicate the traffic condition of that road, where the traffic condition of a road segment $r_{i,j}$ in a given time slot t is denoted as $x_{i,j}^{t}$ and is defined as the mean of the speed of all vehicles driving within the traffic flow on that road segment within the time slot t [23].

(1) $$x_{i,j}^{t}=E\left(\vartheta_{i,j}^{t}\left(v_{i}\right)\right)$$

where $\vartheta_{i,j}^{t}\left(v_{i}\right)$ is the speed of the vehicle $v_{i}$ in road $r_{i,j}$ during time slot t. To estimate the mean speed of traffic flow, the average of all vehicles speed on the road segment during the time slot t is calculated. The average is calculated over different speeds of all vehicles. In this context, we have made the assumption that during each time slot, the traffic condition on a segment is uniform. The traffic statistics of the mean speed for each road segment is calculated through the vehicles’ travel experience reports; it is also estimated using the vehicular traffic measurement at loop detectors in network roads [24].

Let $d_{i,j}$ denote the link delay time, which is the rounded number of time slots required for a vehicle to cross the road segment $r_{i,j}$ that has the length $l_{i,j}$ and the traffic condition $x_{i,j}^{t}$, note that $d_{i,j}$ also includes intersection waiting delay $\omega_{j}$ that vehicles take to exit intersection j before crossing to the next road segment $r_{j,k}$. Thus, the link travel delay for road segment $r_{i,j}$ is computed in (2).

(2) di,jthe=li,jxi,jt+ωj

During each time slot $t_{x}$, each road segment $r_{i,j}$ is associated with a weight, denoted as $w_{i,j}\left(x\right)$, that changes dynamically in each time slot according to the road segments reservations, and it can be computed using (3):

(3) $$w_{i,j}\left(x\right)=C_{i,j}-{Ř}_{i,j}\left(x\right)$$

where: ${Ř}_{i,j}\left(x\right)$: is the number of reserved positions in the road segment $r_{i,j}$ during time slot x. $C_{i,j}$: is the road capacity, which is the maximum number of vehicles that are crossing the road segment $r_{i,j}$ simultaneously without $r_{i,j}$ being congested, formally, let $V_{i,j}=\left(v_{i,j}^{1},v_{i,j}^{2},\ldots ,v_{i,j}^{n}\right)$ be a fleet of vehicles that are crossing the road segment $r_{i,j}$ simultaneously. We say that the road segment $r_{i,j}$ is in congestion condition if (4) is satisfied, in other words, if the difference of the aggregated vehicles link delay time and the delay time of the road $r_{i,j}$ times the number of vehicles in the fleet (n), is greater than or equal to ε:

(4) $$\overset{n}{\underset{i=1}{\sum }}d_{i,j}^{i}-\left(n\times d_{i,j}\right)\leq \varepsilon$$

where $d_{i,j}^{i}$ is the link delay time of vehicle $v_{i,j}^{i}$, and the value of ε is equal to zero in most road segment, except for some special road segments that witness fluctuation in road demand due to special condition. In such cases, ε is evaluated based on the previous statistics of these regular road demand fluctuations. It is important to note that in traffic congestion situations there are two kinds of congestion, recurrent and non-recurrent congestion [25]. The first one arises due to fluctuation in demand and the road conditions, and it is not related to any special incident. While the latter is related to some special conditions caused by breakdowns, accidents and other random events. In DIFTOS, non-recurrent congestion is treated as a special case, to be further discussed in Section 4.8.3.

### 4.4. Road Reservation Matrix

Let Ω be a given set of road segments (5), and T a given set of successive time slots (6), the process of reservation of Ω during T is managed using road reservation matrix, denoted as $M_{\Omega ,T}$, which contains the weights of all the road segments of Ω, during all the time slots in T, since the weights change dynamically based on the road demands, $M_{\Omega ,T}$ is managed and updated by the local DIFTOS server that is responsible for the region that incorporates Ω.

(5) $$\Omega =\left\{r_{0},r_{1},r_{2},\ldots ,r_{N-1}\right\}$$

(6) $$T=\left\{t_{0},t_{1},t_{2},\ldots ,t_{M-1}\right\}$$

(7) $$M_{\Omega ,T}={\left(w_{r,t}\right)}_{m\times n}$$

where $w_{r,t}$ is the weight of the road segment $r_{i,j}$ during time slot t, as defined in (3).

### 4.5. Problem Formulation

Given the source road segment $r_{s}$ and the destination road segment $r_{d}$ the objective of every vehicle v is to determine the shortest path from the source road segment $r_{s}$ to the destination road segment $r_{d}$ with the optimum least congested road segments while respecting the road quota priority reservation. Let $P=\left\{r_{s},\ldots ,r_{d}\right\}$ be the shortest path from $r_{s}$ to $r_{d}$, and $T_{v}=\left\{t_{i},t_{i+1},\ldots ,t_{n}\right\}$ be the estimated required time for v to drive from $r_{s}$ to $r_{d}$. We say that path P is not congested if (8) is satisfied:

(8) $$\forall t_{r}\in T_{v}\;\mathrm{and}\;r\in P\Rightarrow M\left(w_{r,t}\right)>0$$

where $t_{r}$ denote the time slots when the vehicle v crosses the road segment r.

### 4.6. Priority Quota

In DIFTOS, the last available position in a road segment is reserved according to the vehicle’s needs and priorities. In other words, DIFTOS reserves a quota of road capacity for the vehicles whose priority is to use this road, and rerouting them to other roads will cause their travel delay to exceed a predefined threshold.

Let $w_{i,j}^{q}\left(t\right)$ be the quota limit of road $r_{i,j}$ during time slot t. For example, if $C_{i,j}$ = 10 and $w_{i,j}^{q}\left(t\right)$ = 3, that means that the first seven positions in $r_{i,j}$ can be reserved by any vehicle, while the last three positions are reserved only for vehicles that prioritize $r_{i,j}$ in their path. As shown in (9), if the difference of travel delay of $r_{i,j}$ and the delay of the alternative road (or path) after the rerouting is less than the rerouting toleration threshold then $r_{i,j}$ is a priority road for vehicle v (see Algorithm 1).

(9) $$d_{x}^{v}-d_{i,j}^{v}\geq \mu$$

where μ is the rerouting toleration threshold (the threshold is adjusted dynamically according to the road network congestion, the greater congestion, the rerouting toleration threshold increase), and $d_{x}^{v}$ is the delay of the alternative road (or path) after the rerouting.

### 4.7. Shortest Path and Road Rerouting

As mentioned earlier, the objective of DIFTOS is to find for each vehicle the shortest path from its source to the destination point, such that all the road segments’ weights are positive during all the time slots (8). In addition to that, the priority quota of each road segment should be wisely assigned to the legitimate vehicles.

Firstly, the initial shortest path is computed based on the link travel delay with the weight constraint, after that the set of predicted congested road segments within the shortest path is determined. Following that, a rerouting procedure will be applied to each one of these segments or sub-paths formed by the predicted congested road segments (a set of continuously connected road segments).

**Algorithm 1** Priority Quota Constrained Shortest Path1: **Function** Shortest-Path-Quota-Priority ($G,r_{s},r_{d},M$)2: $P_{ini}\leftarrow Weight\_Constrained\_Delay\_Shortest\_Path(G,r_{s}$, $r_{d},M)$3: **for** each road segment $r\in P_{ini}$
**do**4: **if** ($w_{r}\left(t\right)<w_{r}^{q}$) **then**5: $d_{r}^{x}\leftarrow rerout\_delay\left(r\right)$6: **if** ($d_{r}^{x}-d_{r}\geq \mu$) **then**7: Rerout(r)8: **end if**9: **end if**10: **end for**

### 4.8. Path Reservation

The vehicles’ optimal path is determined by DIFTOS servers as follows.

#### 4.8.1. Path Request

**Figure 3:**
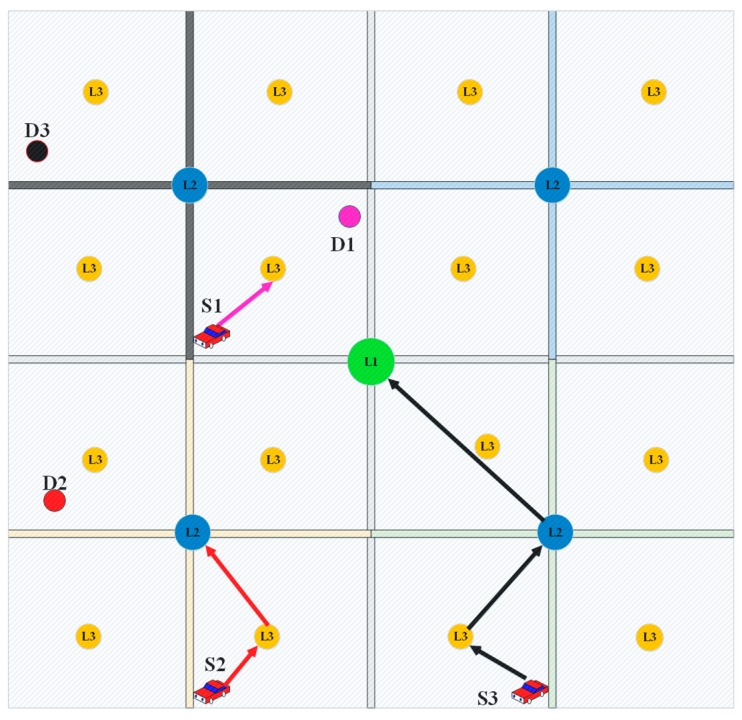
Path request process.

To avoid overloading the upper levels servers, each path request is treated based on its scope, more specifically it is treated at the lowest level that covers its source and destination positions, thus short paths that are within the scope of level 1 server are treated locally. Firstly, the vehicle initiates a path request, by sending a path request packet (PReq) with its current position and the final destination position. The PReq will be sent to the lowest level VVS through V2V communication, in case of isolated node where there are no neighbors in its coverage area, store–carry–forward approach is applied to deliver the path request to the VVS, the latter will decide either to treat the PReq locally or not if the destination position is situated inside its coverage zone (e.g., S1 in Figure 3). Otherwise, the PReq packet is forwarded to the upper-level VVS (e.g., S2 Figure 3). Similarly, long path requests are treated in the highest level VVS (e.g., S3 Figure 3). Upon the reception of the PReq packet, the VVS computes the shortest path from all possible paths which weighs greater than zero at the expected arriving time (fully reserved roads will be excluded from shortest path computing). Taking into consideration the previously answered path requests, and the routes’ weights from the RRM.

#### 4.8.2. Path Reply

After computing the shortest path, the server will send back path reply packet (Prep) to the source vehicle as well as to all the servers which have a coverage area of at least one route segment included in the shortest path, the servers will update their RRM accordingly.

#### 4.8.3. Path Update

In case a vehicle has encountered delay due to some accident or a vehicle has stopped on the way, the vehicle should send path update packet (Pud) to the corresponding servers. If the time gap between the pre-estimated shortest path and the current time is larger than a predefined threshold, the Pud is treated as a PReq and the previous shortest path is ignored. Otherwise, the delay in the shortest path is adjusted accordingly. 

## 5. Simulation

In this section, we present the simulation details of the proposed system. Table 3 shows the software that was used to conduct the simulation.

### 5.1. Map Extraction

The simulation is conducted using a real map of a part of Beijing city. The map is extracted from OpenStreetMap [26]. This is a free open-access website that maintains data about maps and other data such as roads, trails, and railway stations from all over the world. Any map can be easily extracted by specifying the GPS coordinates of the desired area. The raw map data is downloaded in the form of filename.osm file, which is the OpenStreetMap file format. After that, the utilities (netconvert, polyconvert and randomTrips.py) that are part of the SUMO traffic simulator are used to generate the road network file (filesname.net.xml) and the routes file (filename.rou.xml) respectively.

### 5.2. Network Simulation

**Figure 4:**
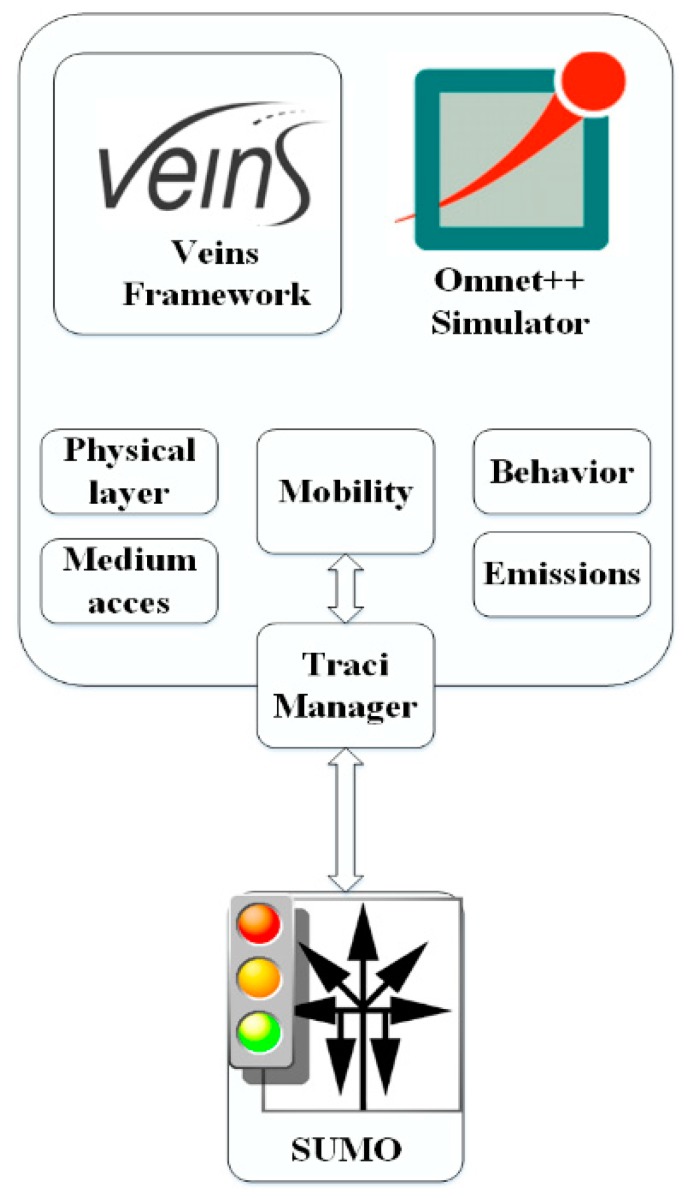
Simulation flow.

The simulation is conducted using Omnet++ network simulator [27] along with the Vehicle simulation framework Veins [28], and the SUMO traffic simulator. During the simulation, three programs are running in parallel: OMNeT++ manages the network simulation, SUMO runs the road traffic simulation and sumo-launched acts as a proxy between the two. SUMO dynamically changes the values through the Traci interface. Veins’s TraciManager class is responsible for connecting Sumo and Omnet++ as shown in Figure 4.

#### V2V Communication Parameters

A detailed description of the wireless communication model used in the simulation is described in Table 4.

## 6. Performance Evaluation

In this section, we evaluate the performance of DIFTOS based on different metrics, and compare it to the baseline system, under different simulation parameters:

### 6.1. Baseline

To evaluate the performance of DIFTOS, we have compared its performance with the following baselines:

CD: Since most of the recent state-of-the-art navigation systems in the industry [2,3,4,5] are using Dijkstra’s shortest path algorithm based on real-time congestion statistics. In addition to other state-of-the-art works, such as [15,29], compared their systems with Dijkstra based congestion avoidance system as a benchmark. Therefore, we have implemented a centralized system and used it as a comparing baseline; we will refer to it as CD (Centralized Dijkstra).

SAINT: The system presented in [15], where a centralized traffic control system maintains the road reservation matrix. The vehicles solicit the server to get the least congestion path to their destination based on the road reservation matrix (Unlike CD, which does not consider road reservations).

DIVERT: The hybrid system presented in [16], where the path calculation is computed by a centralized server, and some of the computing is done by the vehicles (similar to DIFTOS approach). However, a preinstalled infrastructure is required (RSU, cellular network). Unlike DIFTOS, there are not hierarchical levels of the vehicular servers.

### 6.2. Metrics

- Request Round Trip Time (RRTT): The time required to send a path request packet and receive the path reply.
- Computation Cost (CC): The number of operations the server performs to determine the optimal path for a given RReq and maintain the road reservations.
- Trip Time (TT): The time required by a vehicle to reach its destination after receiving the RRep packet from the server.
- Traffic Messages (TM): The number of exchanged messages to compute and maintain the optimal path. In this context, only the traffic related messages are counted, such as route request messages, server election messages, route reply messages and route update messages.

### 6.3. Evaluation Parameters

In the performance evaluation, we will investigate the impact of the following parameter on the system performance:

Traffic Density (TD): The number of vehicles per square kilometer involved in the simulation. By increasing this parameter, we can evaluate the scalability of the system under heavy traffic conditions. We have conducted the simulation by changing the traffic density to different values, and observing its effect on the performance metrics (RRTT, CC, TT) with each new value. We have used the following values: 50, 100, 200, 500, 1000 and 5000 (vehicles/km^2^). Accidents Count (AC): The number of accidents during the simulation. By increasing this parameter, we can evaluate the robustness of the system against traffic accidents, and its ability to mitigate the effects of such accidents. We have increased the number of accidents each time and observed its effect on the performance metrics (RRTT, CC, TT). We have used the following values: 5, 10, 20, 30, 40 and 50 accidents. 

The presented results in the next subsection are the average of the 100 simulations run for each value of the evaluation parameters.

### 6.4. Results Analysis

**Figure 5:**
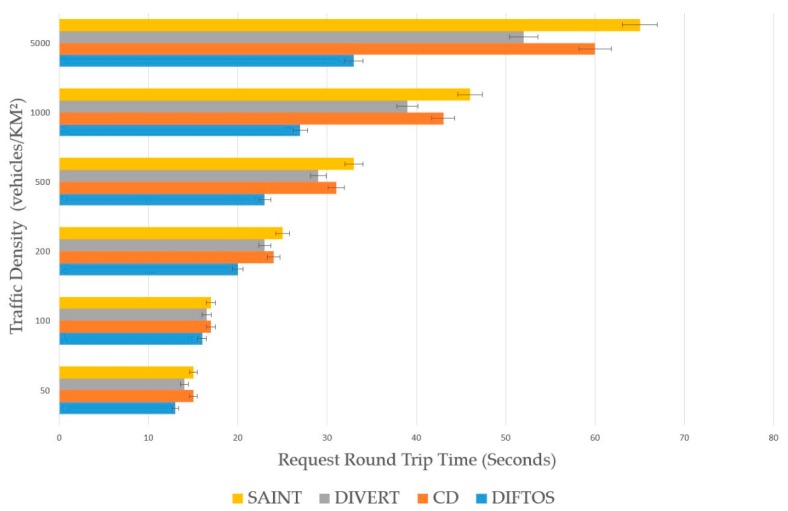
Traffic density vs request round trip time.

The performance of the four systems in terms of RRTT in different traffic densities is presented in Figure 5. We can observe that DIFTOS has the shortest RRTT, especially when the traffic density increases, that is due to the centralized nature of SAINT and CD (partially centralized in case of DIVERT). In CD and SAINT, the average RRTT time increases exponentially with the increase of TD, which is because the centralized server gets busy when more RReq arrive simultaneously. Which is not the case with DIFTOS, because some of the RReq are treated by lower level servers and do not need to be forwarded to the high-level server. Thus, DIFTOS sustains its stability in terms of RRTT, even when the TD is very high (see Figure 5). DIVERT also maintains a relatively short RRTT compared to SAINT and CD, but longer RRTT compared to DIFTOS. That is because the vehicular servers are not organized as a hierarchy like DIFTOS, therefore local RReq need to travel long distances.

**Figure 6:**
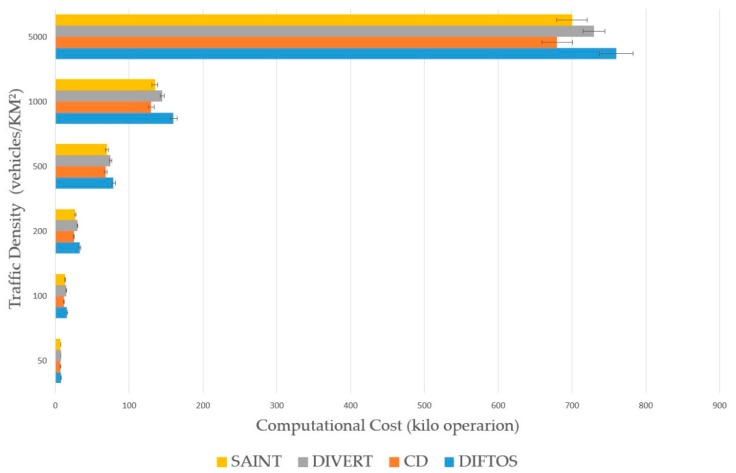
Traffic density vs. computational cost.

Figure 6 presents the performance of the four systems in terms of CC in different traffic densities. CD has the least CC among the four systems, that is because CD does not maintain the reservation matrix as the other three systems, it only calculates the optimal path for each vehicle regardless of the traffic conditions. SAINT and DIVERT have similar CC in different densities, and their CC is higher than CD’s; this is because they maintain a road reservation matrix, which needs regular updates that require more computation. From this shallow point of view, one could argue that CD, SAINT and DIVERT perform better than DIFTOS in terms of CC, but the calculation in these systems is done in the centralized server (partial at vehicles in DIVERT), while in DIFTOS the computation is distributed among the VVS; thus, the values of CC shown in Figure 6 are the sum of all VVS in case of DIFTOS.

**Figure 7:**
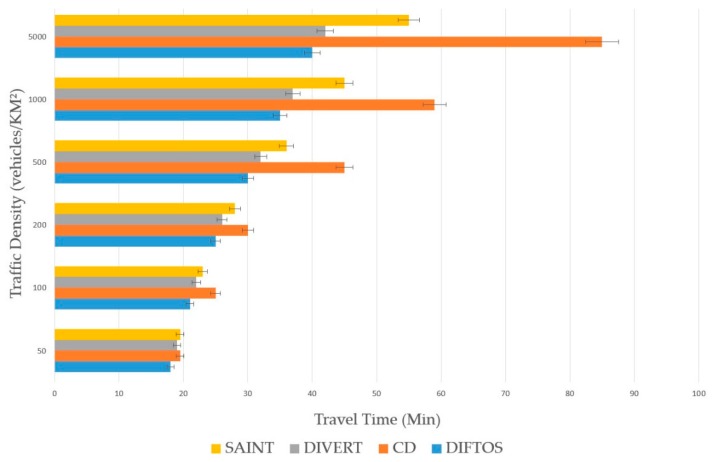
Traffic density vs. travel time.

The effectiveness of DIFTOS in terms of travel time is obvious in Figure 7, where DIFTOS achieves the shortest average travel time. DIFTOS could maintain a reasonable TT, even under high traffic density. That is due to the re-routing strategy that allows it to determine the expected congested roads, and re-route vehicles away from these roads before they arrive. DIVERT also achieved a relatively short average TT for the same reason mentioned above. SAINT’s average TT is considerably longer than DIFTOS and DIVERT’s. However, CD had the longest average TT; this is because it does not consider the road reservations when computing the optimal paths.

**Figure 8:**
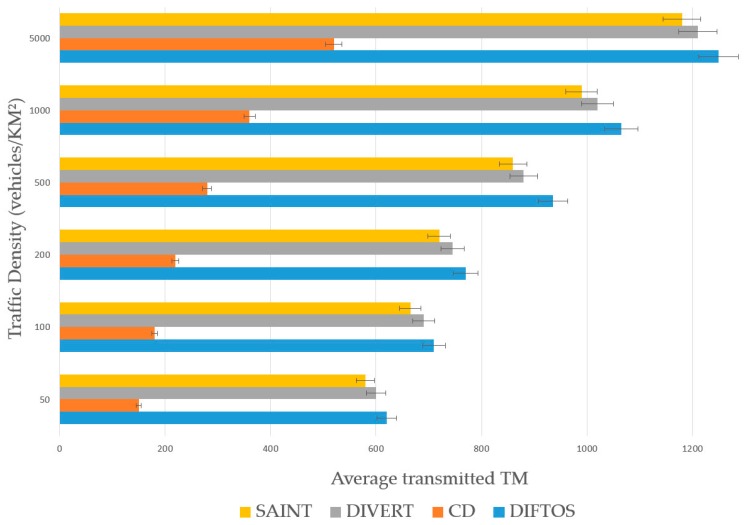
Traffic density vs. communication overhead.

The traffic related communication overhead of the four systems is presented in Figure 8. CD had the least amount TM, which is due to its static nature; once the optimal path is determined, the vehicles do not send any update to the server. SAINT, DIVERT and DIFTOS send much more TM compared to CD, which is because they all maintain the road reservation matrix, thus the vehicles in these systems need to send a route update about their position to the server to update the road reservation matrix. DIFTOS’s communication overhead is slightly higher than SAINT and DIVERT’s, which is because the servers in DIFTOS are designed in a hierarchical manner, and the low level servers need to update their higher servers regularly.

**Figure 9:**
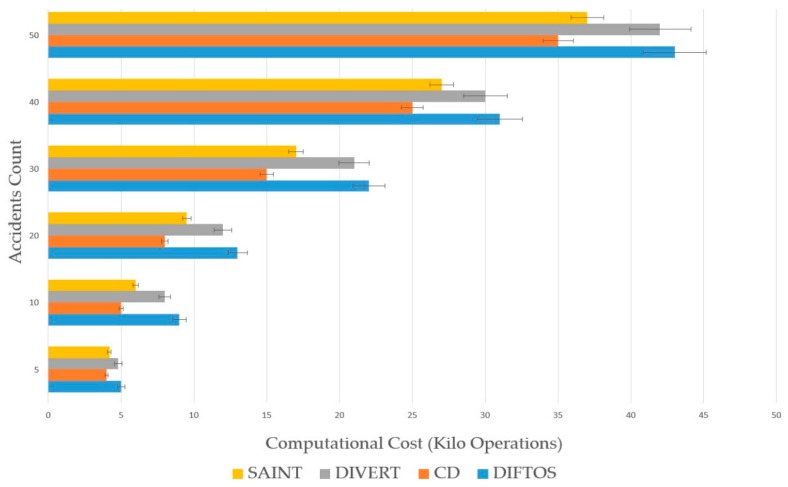
Accident count vs. computational cost.

**Figure 10:**
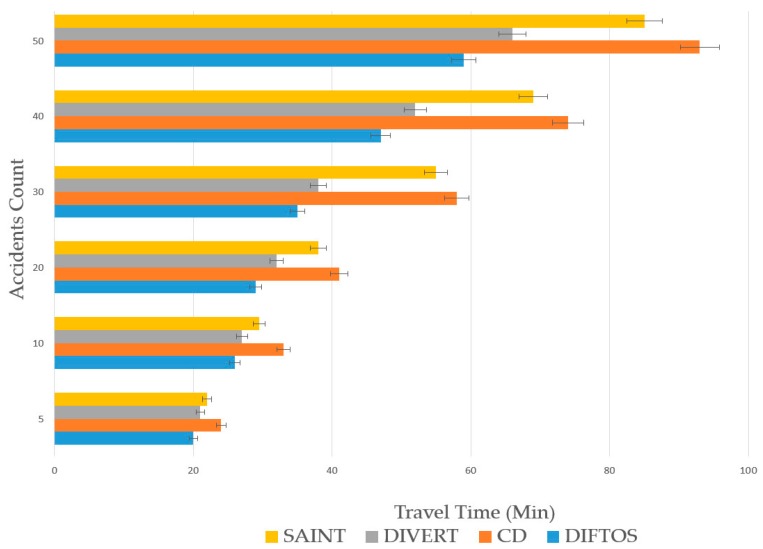
Accident count vs. travel time.

The effect of accidents on the computational cost and average travel time is presented in Figure 9 and Figure 10. The number of accidents is increased each time to evaluate the robustness of the four systems against traffic accidents, and their ability to mitigate the effects of such accidents. CD had the best resistance against accidents in term of CC. The reason for this is that in case of congestion due to an accident, the vehicles follow the same congested paths, and do not request the server for a new path, thus no additional computation is required. However, the CD’s average TT is the longest among the four systems, due to the aforementioned reason. SAINT and DIVERT have shorter TT compare to CD, as they update the vehicles’ paths in case of an accident. DIFTOS achieves the shortest TT among all systems. This is because the path update in case of an accident can be resolved at the local server level and it does not need to travel to a centralized traffic center, like in SAINT and DIVERT.

## 7. Conclusions

In this paper, we have presented a new distributed infrastructure-free congestion avoidance and traffic optimization system for VANET named DIFTOS. The proposed system is completely infrastructure-free and does not rely on any external infrastructure such as roadside units (RSU) or 3G/4G networks. We have proved by means of simulation the scalability and robustness of DIFTOS in different traffic conditions.

Future Directions: The simulation results prove the effectiveness, robustness, and scalability of DIFTOS. However, DIFTOS still needs many improvements, in this section, we will list our future directions to improve DIFTOS:

- The inter-vehicle communications are encrypted. However, as long as vehicles’ traces are fully disclosed, the user’s identity in some cases can be inferred even if pseudonyms are used. Therefore, DIFTOS still needs to be improved from the privacy point of view. Enhancing DIFTOS with an additional security and privacy framework is one of our future research directions.
- DIFTOS’s distributed architecture improves the scalability of the system and reduces the load on servers, as the path requests are treated as different servers. However, at upper-level servers, it becomes difficult for a single vehicle to maintain the RRM, due to the large size of data, and the huge amount of computation needed to resolve many path requests simultaneously. Therefore, optimizing DIFTOS to distribute the server calculation as a vehicular cloud server, where many vehicles collaborate to maintain RRM, is one of our future directions.
- The current hierarchical partition still needs improvements, as cross levels paths still need to solicit upper levels servers. In our next work, we will try to change the path request procedure, where two adjacent servers of the same level can cooperate to solve a path request without the need to forward it to higher level servers.

## Figures and Tables

**Table 1:** Comparison between DIFTOS and state-of-the-art systems.

System	Infrastructure Dependency	Server Architecture	Server Design	Congestion Treatment Policy
DIFTOS	Infrastructure-Less	Distributed	Hierarchy of Vehicular Servers (Vehicles)	Path requests for roads reservation (Congestion will never occur)
SAINT [15]	RSU + Cellular network	Centralized	Traffic Center (Computer)	Traffic estimation (Possible congestion)
DIVERT [16]	Cellular network	Partially Distributed	Traffic Center + Vehicular Servers (Computer + Vehicles)	Congestion detection (Possible congestion)
RTP [13]	RSU + Cellular network	Centralized	Traffic Center (Computer)	Congestion mitigation based on path planning (Possible congestion)
NRR [14]	RSU + Cellular network	Centralized	Traffic Center (Computer)	Heuristic rerouting to avoid congestion (Possible congestion)
RoadRunner [17]	Cellular network	Distributed	Mobile app + Centralized Server (Computer + smart phones)	Tokens for road reservation (Congestion will never occur)

**Table 2:** Notations summary.

Symbol	Description
G=(I,Ω,V)	Road network graph that represents the city map
I	Set of all vertices in the road network graph
Ω	Set of all edges in the road network graph
V	Set of all vehicles in the city
$r_{i,j}$	The road segment from intersection i to intersection j
$T=\left\{t_{1},t_{2},\ldots ,t_{n}\right\}$	Set of successive time slots
$x_{i,j}^{t}$	The mean of the speed of all vehicles driving within the traffic flow on the road segment $r_{i,j}$ within the time slot t
$\vartheta_{i,j}^{t}\left(v_{i}\right)$	The velocity of the vehicle $v_{i}$ in road segment $r_{i,j}$ during time slot *t*
$d_{i,j}$	Travel delay required to cross road segment $r_{i,j}$
$l_{i,j}$	The length of the road segment $r_{i,j}$
$\omega_{j}$	The waiting delay at intersection j
$C_{i,j}$	The capacity of the road segment $r_{i,j}$
${Ř}_{i,j}\left(t\right)$	The number of available positions in the road segment $r_{i,j}$ during time slot t.
$w_{i,j}\left(t\right)$	The weight of road segment $r_{i,j}$ during time slot *t*
$P=\left\{r_{s},\ldots ,r_{d}\right\}$	The path yielded by connecting the road segments from $r_{s}$ to $r_{d}$
$M_{\Omega ,T}$	Road reservation matrix of the road set Ω during the period T
$w_{i,j}^{q}\left(t\right)$	The quota limit of the road $r_{i,j}$ during time slot t

**Table 3:** Simulation parameters.

Parameter	Description
Network simulator	Omnet++ 5
Traffic simulator	SUMO 0.27.1
Map source	Open street map
Simulated location	Part of Beijing city, China
Simulated area	10 km × 10 km

**Table 4:** Wireless communication parameter.

Parameter	Value
PHY model	802.11 p
Channel frequency	5.890 × 10^9^ Hz
Propagation model	Two ray
MAC model	EDCA
Propagation distance	450 m
Maximum hop	15
Fading model	Jakes model rayleigh fading
Shadowing model	LogNormal
Antenna model	Omnidirectional
Transmission power	20 mW
